# Prevalence of tuberculosis in Rwanda: Results of the first nationwide survey in 2012 yielded important lessons for TB control

**DOI:** 10.1371/journal.pone.0231372

**Published:** 2020-04-23

**Authors:** Patrick Migambi, Michel Gasana, Claude Bernard Uwizeye, Eliane Kamanzi, Vedaste Ndahindwa, Nico Kalisvaart, Eveline Klinkenberg

**Affiliations:** 1 Rwanda Ministry of Health / Rwanda Biomedical Centre, Kigali, Rwanda; 2 Family Health International, Kigali, Rwanda; 3 School of Public Health, College of Medicine and Health Sciences, University of Rwanda, Kigali, Rwanda; 4 KNCV Tuberculosis Foundation, The Hague, the Netherlands; 5 Department of Global Health and Amsterdam Institute for Global Health and Development, Amsterdam University Medical Centers, Amsterdam, The Netherlands; Public Health England, UNITED KINGDOM

## Abstract

**Background:**

Rwanda conducted a national tuberculosis (TB) prevalence survey to determine the magnitude of TB in the country and determine to what extent the national surveillance system captures all TB cases. In addition we measured the patient diagnostic rate, comparing the measured TB burden data with the routine surveillance data to gain insight into how well key population groups are being detected.

**Methods:**

A national representative nationwide cross-sectional survey was conducted in 73 clusters in 2012 whereby all enrolled participants (residents aged 15 years and above) were systematically screened for TB by symptoms and chest X-ray (CXR). Those with either clinical symptoms (cough of any duration) and/or CXR abnormalities suggestive of TB disease were requested to provide two sputum samples (one spot and one morning) for smear examination and solid culture.

**Results:**

Of the 45,058 eligible participants, 43,779 were enrolled in the survey. Participation rate was high at 95.7% with 99.8% of participants undergoing both screening procedures and 99.0% of those eligible for sputum examination submitting at least one sputum sample. Forty cases of prevalent mycobacterium tuberculosis (MTB) and 16 mycobacteria other than tuberculosis (MOTT) cases were detected during the survey. Chest x-ray as screening tool had 3 and 5 times greater predictive odds for smear positive and bacteriological confirmed TB than symptom screening alone respectively. A TB prevalence of 74.1 (95% CI 48.3–99.3) per 100,000 adult population for smear positive TB and 119.3 (95% CI 78.8–159.9) per 100,000 adult population for bacteriological confirmed MTB was estimated for Rwanda.

**Conclusions:**

The survey findings indicated a lower TB prevalence than previously estimated by WHO providing key lessons for national TB control, calling for more sensitive screening and diagnostic tools and a focus on key populations. Use of chest x-ray as screening tool was introduced to improve the diagnostic yield of TB.

## Introduction

In 2012, the World Health Organization (WHO) estimated the global tuberculosis (TB) prevalence rate to be 166 per 100,000 population and the incidence rate to be 122 per 100,000 population while for the African region the estimated rates were 303 and 255 respectively. For Rwanda, the TB prevalence and incidence rates were estimated at 114 and 86 per 100,000 population respectively. In that year, Rwanda notified 6,208 TB cases, 63.3% of the 9,800 estimated incident TB cases [1]. The observed disparity raised the question whether the disease exists at lower levels than estimated by WHO or whether the observed gap results from low TB case detection or underreporting of TB cases. Elsewhere, both hypotheses have been demonstrated to be plausible [2].

If prevalent cases outnumber reported cases, as observed in some TB prevalence surveys [3–5], it can be inferred that not all people are captured in the surveillance system [4–6], either because they do not seek [3] or delay to seek care when they experience TB symptoms. It could also be inferred that they are not diagnosed by the routine detection approaches [5, 7] or not reported when diagnosed [8]. If prevalent cases are below WHO estimates [2], this could mean the TB program is performing better than pictured.

Rwanda never conducted a TB prevalence survey. Previous estimations on the TB burden in Rwanda were based on WHO modelling. To obtain a more accurate estimate of the national TB burden, Rwanda undertook a national representative survey to directly measure the TB prevalence as well as the patient diagnostic rate to determine the capacity of the surveillance system to capture TB cases [9]

## Methods

### Survey design, population and sampling

A cross-sectional population-based survey covering the whole of Rwanda was designed using a cluster sampling approach proportionate to population size. The survey was designed using the latest guidance of WHO at the time as outlined in the ‘lime book’ [10]. All residents of selected villages who had been living in the household for at least 1 month prior to the interview and who were aged 15 years and above were eligible for the survey, including bed-ridden patients, pregnant women and people on TB treatment during the survey.

Persons mentally challenged and unable to provide adequate responses and people within institutions (prisons, etc.) were not included. Field data collection was conducted from March to December 2012.

At the time of survey planning, a TB prevalence of 252/100,000 was projected for Rwanda by WHO [11]. Expert opinion in country felt that the burden estimations by WHO could be “overestimated” and the true burden in the country was actually lower. Therefore, 70% of the burden estimate was used as basis for the sample size calculation. Using an anticipated prevalence of 176/100,000 population for sputum smear positive (SS+) pulmonary in the adult population of Rwanda, a participation rate of 95%, a relative precision of 23%, a confidence level of 95%, and an estimated design effect of 1.7, we calculated a survey sample size of 42,598 adults. Administrative sectors were sampled proportionally to the size of their populations. A simple random sampling of villages (Umudugudu) within selected sectors was carried out. Based on operational feasibility, the number of clusters acceptable for the survey was 70, resulting in a targeted sampled size of 610 persons per cluster. Kigali has a substantially higher caseload than the other provinces and harbors around one quarter of all pulmonary TB cases in Rwanda. To obtain a more precise estimate for Kigali it was decided to select three additional cluster in Kigali. Therefore during analysis weighing was applied to balance the contribution of the Kigali clusters. With the three additional clusters in Kigali the total number of clusters was 73 and the total targeted sample size 44,500 adults.

### Tuberculosis screening and diagnosis strategies

The screening strategy followed the WHO recommended approach whereby all enrolled participants were administered short-structured symptoms interview followed by a chest X-ray (CXR). Those persons with either clinical symptoms (presence of cough of any duration) and/or CXR abnormalities suggestive of TB disease were eligible to provide two sputum samples (one spot and one morning) for smear examination and solid culture. In addition, those who refused to be screened by CXR were requested to provide sputum, irrespective of symptoms. Sputum sample transportation was organized twice a day from the survey field site to the nearest health center laboratory. There, upon arrival of the samples the survey lab technician prepared slides of all collected sputum samples using Auramine staining technique. All slides were read by fluorescence microscopy for presence of acid-fast bacilli (AFB). After slide preparation, the remnant of the sputum samples was packed and sent within 24 hours to the National Reference Laboratory (NRL). At NRL, concentrated smear, solid culture (Löwenstein–Jensen medium), MTB identification (Capilla SD bioline test) and TB drug susceptibility testing (DST) was performed on all sputum samples received from the field. In addition, the NRL performed quality control of the field slides after re-staining using Auramine technique microscopy. All those eligible for sputum were offered HIV counselling and testing on site in the field via rapid testing (determine and unigold) as per the national algorithm.

### Key variables definition

A sputum smear (s) was considered positive if there was at least one AFB per 100 immersion fields. For all positive slides the bacilli load was quantified (Zero AFB/ 1 length; 1–19 AFB /1 length; 20–199 AFB /1 length; 5–50 AFB/1 field, on average,>50 AFB/1 field, on average). A slide was considered as negative if no AFB was detected after reading of at least 300 immersion fields. A culture (C) was considered positive if, after culture on a solid medium, there was isolation of any number of *Mycobacterium Tuberculosis* complex colonies as confirmed by Capilla test [10]. No growth or a negative culture was defined as not showing growth after 56 days in the incubator.

To define the outcome indicators for analysis, the following internationally accepted survey case definitions as outlined in the guidelines for prevalence surveys [10] were used: A definite survey TB case (bacteriologically-confirmed survey TB case) if there was presence of one positive specimen for MTB (CTB) on culture and at least one of the following conditions: AFB-S positive (smear-positive) or positive culture in another specimen or a chest X-ray abnormal finding in lung at central audited reading. An AFB-S positive survey TB case (smear-positive TB case), if one AFB-S positive specimen and at least one of the following conditions: a CTB positive (definite survey TB case), or an AFB-S positive in another specimen but not CTB positive and no isolation of *Mycobacterium Other Than Tuberculosis* (MOTT) (probable TB case) or a chest X-ray abnormal finding in lung at central reading but not CTB positive and no isolation of MOTT (probable TB case).

### Data management and analysis

Data collection was paper based and data entry was done in Epidata 3.1. Randomly, 10% of each questionnaire was double entered by two different data entry clerks. A maximum of 1% of errors was allowed. If error was more than 1%, double data entry was conducted. In particular records of all individuals with positive laboratory results (TB and/or HIV) or positive central X-ray readings were cross-checked again one by one with the original forms and registers to correct any inconsistences. The following forms and register were entered electronically: census register and socio-economic status questionnaire, screening questionnaire, questionnaire for participants eligible for sputum examination, field laboratory and NRL TB register, CXR field register and central CXR re-reading result, and risk factors questionnaires. Data cleaning and validation was conducted in SPSS 16.0 including checks for correct merging using the Personal Identification Numbers (PINs).

STATA software package version 11 was used to analyze data. The first stage in the analysis focused on describing eligibility, enrollment and participations by age, sex, province and wealth quintile. Subsequently the outcome of screening (interview and x-ray) and sputum testing was described, disaggregating by key characteristics (i.e. sex, age, type of symptoms or x-ray abnormalities). The core analysis focused on determining the prevalence of pulmonary TB. Analysis followed the global guidance for analyzing national TB prevalence survey [12]. As a first step, a simple cluster level analysis was done whereby the prevalence rates were calculated for each cluster and then combined to one single point estimate with confidence boundaries. The second step was individual level analysis using three modelling approaches that differed in the way missing data was accounted for. The final model as per global guidance is a model applying missing value imputation for those eligible for sputum with missing outcome data and inverse probability weighting to represent all survey participants.

The survey was designed to measure bacteriological confirmed pulmonary TB in the population aged 15 years and above.

We also calculated the patient diagnostic rate, a rate indicating at which level prevalent cases are detected by the routine TB control program. It was determined for smear positive TB cases as the number of reported case per 100,000 persons per year divided by the smear positive prevalence per 100,000 [9].

### Ethics statement

This survey received approvals from the Rwanda National Ethics Committee (RNEC) and a study visa was issued by the National Institute of Statistics of Rwanda (NISR). In addition, Non-Research Determination (NRD) approvals were obtained from the Division of TB elimination (DTBE) and the Division of Global HIV/AIDS (DGHA) of CDC. The survey protocol was also reviewed, approved and monitored by the WHO Global Task Force on TB Impact measurement. During the survey, all eligible persons received comprehensive information on the objectives of the survey, its processes, the use of the collected samples (sputum) and information about x-ray radiation as well as on implications related to their participation. All those participating provided written consent before commencing survey procedures. During X-ray taking all participants were provided with gonal shielding. Respondents diagnosed with TB disease were informed about their result and referred to TB care and treatment center serving the investigated village for TB treatment initiation as per national guidelines. As per national TB program guidelines at the time of the survey, all presumptive TB cases were offered HIV counseling and testing. Those testing HIV positives were referred for proper HIV care and treatment and those testing negative referred for prevention services as per national guidelines.

## Results

### Census, eligibility, participation, screening and sputum examination

**Fig 1:**
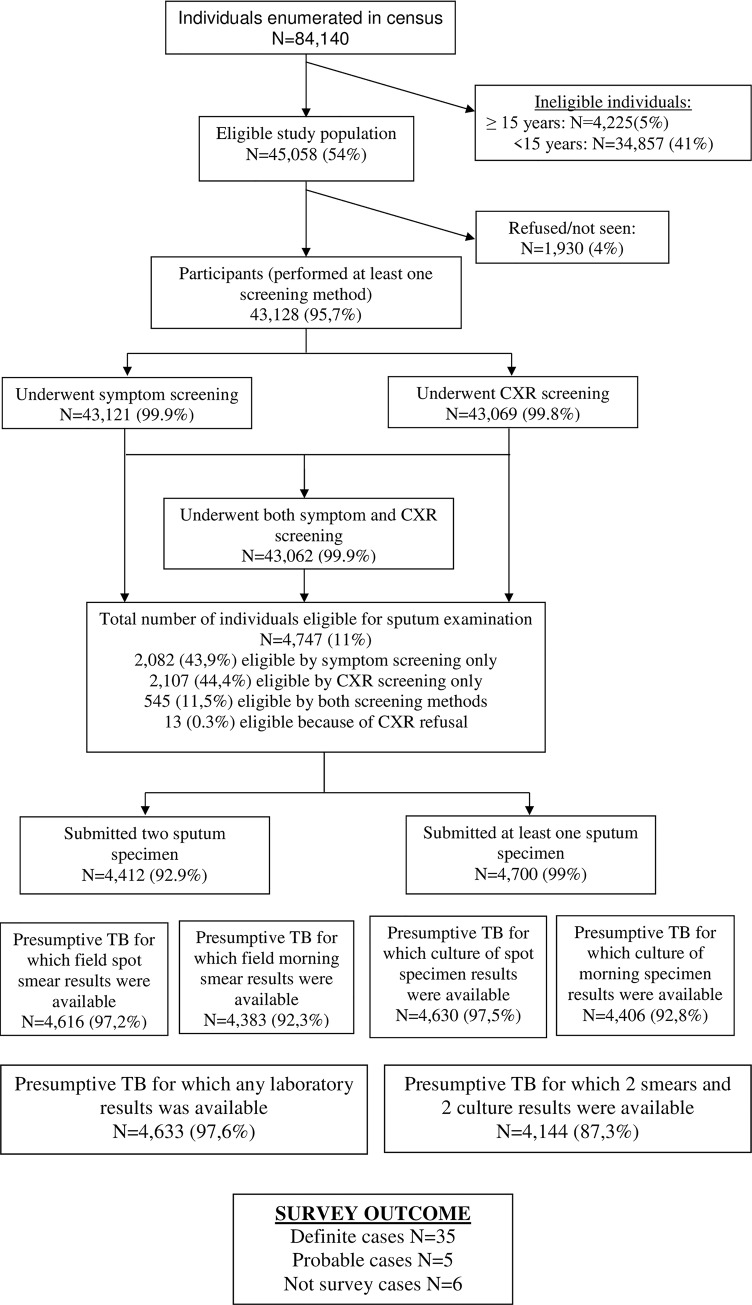
Participants flow diagram of the Rwanda national TB prevalence survey, 2012.

A total of 84,140 individuals were enumerated of whom 45,058 (54%) were eligible individuals invited to participate in the survey. Of them, 43,779 (97%) were enrolled. Of them 43,121 (99.9%) completed the symptom screening interview and 43,069 (99.8%) underwent chest x-ray screening. The overall participation rate was 95.7% [Fig 1].

Of all participants who underwent the screening procedures 4,747 (11.0%) were eligible for sputum examination, 43,9% of them based on symptom screening only, 44,4% based on CXR screening only, 11,5% based on both screening methods and 0.3% because they refused CXR [Fig 1].

Of the 4,747 eligible for sputum examination, 99% provided at least one sputum specimen and 93% provided two sputum specimens. For 87% of those who were sputum eligible, both smear and both culture results were available. [Fig 1].

After review by the medical panel a total of 46 cases with bacteriological indications were considered programmatic TB cases and put on treatment. Applying the survey TB case definitions, 40 cases were identified, 35 definite and 5 probable cases. Six cases were not considered survey cases as they had only an indication of TB in one of the collected samples which was not confirmed by an indication in another sample nor in the central reading of the CXR. [Fig 1].Of the 54 culture positive cases, 38 cases (70%) were identified as Mycobacterium Tuberculosis (MTB) species and 16 (30%) were Mycobacterium other than Tuberculosis (MOTT). Smear microscopy identified 22 (40%) of the 54 cases. None of the 54 culture positive cases was co-infected with both MTB and MOTT.

Eligibility by CXR screening had 3 and 5 times greater predictive odds of smear positive and bacteriological confirmed TB than symptom screening alone respectively. Combining both symptom and CXR screening had even 15 times greater predictive odds for smear positive and bacteriological confirmed TB than symptom screening alone [Table 1].

**Table 1:** Comparing the diagnostic performance of screening approaches for smear positive or bacteriologically-confirmed tuberculosis, Rwanda 2012.

	Number of positive	Odds Ratio	SE	p-value	[95% CI]	
**Positivity of combined field microscopy**						
Symptom screening only (n = 2,092)	4	1.00				
CXR screening only (n = 2,110)	13	3.23	1.85	0.040	1.05	9.94
Symptom and CXR screening (n = 545)	12	11.75	6.80	0.000	3.78	36.58
**Bacteriologically confirmed TB**						
Symptom screening only (n = 2,092)	4	1.00				
CXR screening only (n = 2,110)	20	4.99	2.74	0.003	1.70	14.64
Symptom and CXR screening (n = 545)	14	13.76	7.83	0.000	4.51	41.98

SE, standard error; CI, confidence interval; CXR, chest X-ray; TB, Tuberculosis

### TB prevalence estimates

**Fig 2:**
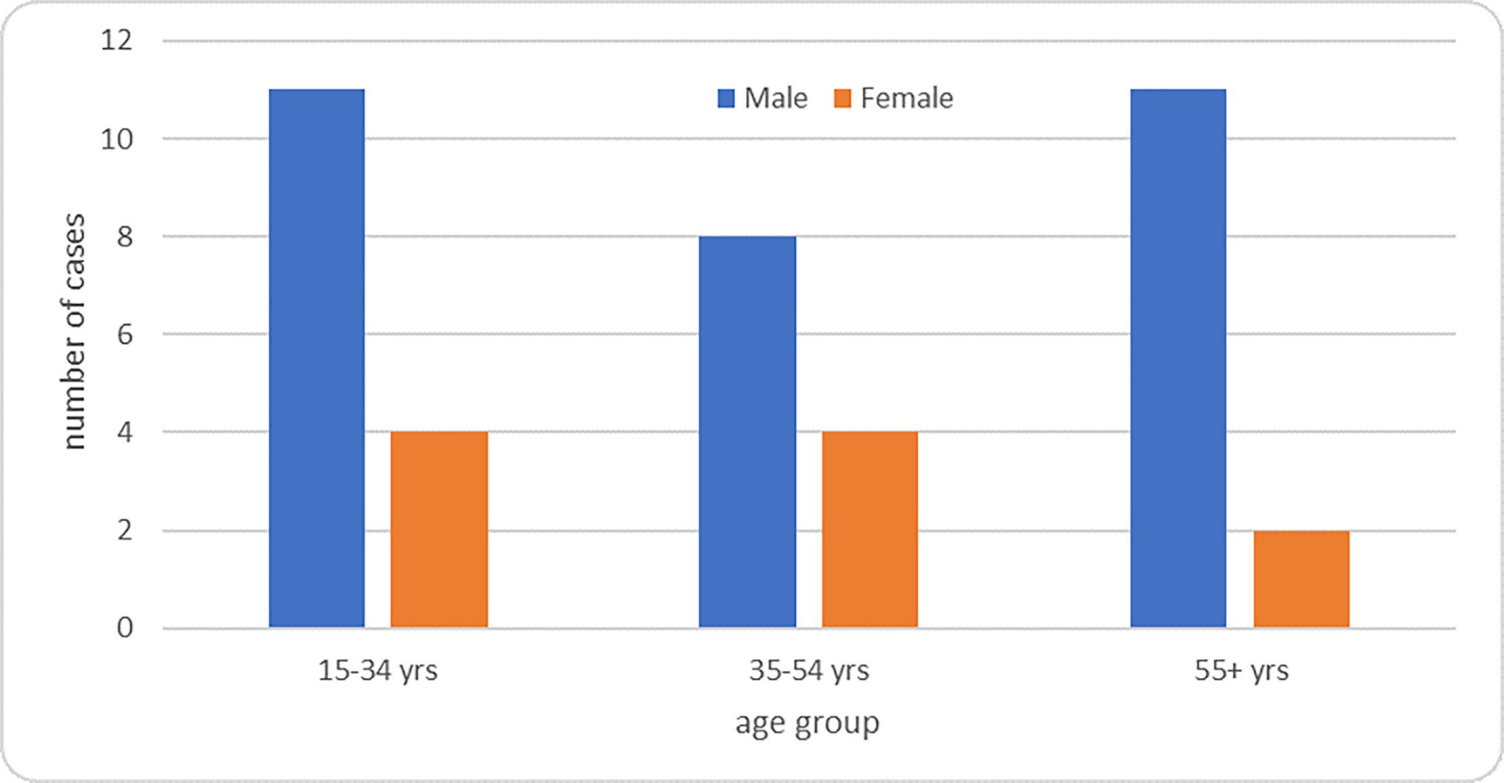
Distribution of diagnosed TB prevalent cases in the survey by sex and age group, Rwanda 2012.

Applying the survey definitions of prevalent cases, 35 definite cases and 5 probable cases were identified. Of the 40 survey cases, 27 (68%) were smear-positive TB and 13 (32%) smear negative TB. Three-quarter (30/40) of the survey cases were male and male cases were predominant in in each age groups [Fig 2].

Of the 40 survey cases only 1 was HIV positive while 4 refused HIV testing. Two of the 40 detected cases were already on TB treatment, while 3 had received previous treatment for TB [Table 2].

**Table 2:** Characteristic of participant and number of adult pulmonary Tuberculosis prevalence by sex, by age, by wealth level, by province, by history of Tuberculosis and by HIV status, Rwanda 2012.

Characteristic	Number of participants*	Number of prevalent TB cases detected
**Overall**	43,128	40
**By sex (**43,128)		
Male	18,195 (42.2%)	30
Female	24,933 (57.8%)	10
**By age group (**43,128)		
15–34 years	24,521 (56.8%)	15
35–54 years	12,229 (28.4%)	12
54+ years	6,378 (14.8%)	13
**Wealth level**^**#**^ **(n =** 43,128)		
Lowest	14,638(42.2%)	13
Middle	14,742(42.2%)	12
Highest	13,684(42.2%)	15
**By Province (n =** 43,128)		
Eastern	9,531(22.1%)	12
Kigali City	5,469(12.7%)	7
Northern	7,135(16.5%)	5
Southern	10,806(25.1%)	6
Western	10,187(23.6%)	10
**By TB history (n =** 43,128)		
Yes	545(1.2%)	3
No	42,561(98.7%)	37
Unknown	22(0.1%)	0
By HIV Status* **(n =** 4,747)		
Positive	218(4.6%)	1
Negative	4,227(89%)	35
Not tested	302(6.4%)	4

* HIV testing was not done for all participant but only for TB presumptive

As per global guidance (ref 12) model 3 was considered the most optimal as it takes into account both the distribution of the enumerated versus the participants as well as missing value imputation for those eligible for sputum examination who are missing a final outcome. Therefore, the weighted prevalence of smear positive TB in the adult population in Rwanda was estimated as 74.1 (48.3–99.3) per 100,000 adults, while the estimated prevalence of bacteriological confirmed MTB was 119.3 (78.8–159.9) per 100,000 adult population. Although the presented point prevalence seems to differ, the figures presented do not differ statistically due to low study power and as you can see the confidence bounds fully overlap so there is no statistical difference by wealth index[Table 3].

**Table 3:** Adult pulmonary Tuberculosis prevalence by age, by sex, by wealth level for model 3 analysis for smear positive and bacteriological confirmed TB, Rwanda 2012.

Characteristic	Prevalence of smear positive TB per 100,000 adult population		Prevalence of bacteriologically confirmed MTB per 100,000 adult population	
	Estimate	95% CI	Estimate	95% CI
**Overall**	74.1	48.3–99.3	119.3	78.8–159.9
**By sex**				
Male	141.9	87.5–196.2	208.2	138.7–277.8
Female	23.7	4.7–42.6	53.0	19.9–86.1
**By age group**				
15–34 years	56.8	27.4–86.2	85.5	46.1–124.9
35–54 years	65.6	21.1–110.2	113.8	35.0–192.6
54+ years	158.8	54.1–263.0	262.4	104.4–420.5
**Wealth level**^**#**^				
Lowest	52.4	16.4–88.5	119.7	54.6–184.8
Middle	68.4	20.7–116.3	96.1	45.9–146.3
Highest	103.0	47.7–158.4	144.4	78.1–210.8

* Those who completed all screening procedures n = 43,128

# for 64 persons the socio-economic questionnaire was not complete and no wealth level could be assigned

Observed prevalence was 4–6 time higher for males than for females for both smear and bacteriological confirmed TB [Table 3]. Observed TB prevalence was highest in the oldest age group of 55 years and above (numerically 2–3 times higher than the other age groups), for both males and female [Table 4].

**Table 4:** Patient diagnostic rate for smear positive adult population in Rwanda, Rwanda 2012.

Characteristics	Smear positive cases reported	Population	Notification rate of smear positive TB per 100,000 population	Estimated smear positive TB prevalence per 100,000 population	Patient diagnostic rate (for smear positive TB)
**Overall**	3,524*	6,187,890	56.9	74.1 (48.3–99.3)	0.77 (0.57–1.18)
**By sex**					
Male	2,331	2,915,958	79.9	141.9 (87.5–196.2)	0.56 (0.41–0.91)
Female	1,169	3,271,932	35.7	23.7 (4.7–42.6)	1.51 (0.84–7.60)
**By age group**					
15–34 years	1,863	3,830,438	48.6	56.8 (27.4–86.2)	0.86 (0.56–1.78)
35–54 years	1,175	1,604,391	73.2	65.6 (21.1–110.2)	1.12 (0.66–3.47)
54+ years	462	753,061	61.3	158.8 (54.1–263.0)	0.39 (0.23–1.13)

Combining the obtained TB prevalence result with the notification figures of 2012, resulted in an overall patient diagnostic rate (PDR) of 0.77 (95% CI 0.57–1.18), with males and those 55 years and above having a lower PDR. [Table 3].

## Discussion

The first National TB prevalence survey in Rwanda conducted in 2012 revealed that the smear positive TB prevalence survey was estimated at 74.1(95% CI 48.8–99.3) per 100,000 adult population and bacteriological confirmed TB at 119. (95% CI 78.8–159.9) per 100,000 adult population. The participation rate was at 95.7% and sputum collection rate of 99.0%.

Contrary to several other countries where national TB prevalence surveys have been recently completed and where prevalence rates were higher than expected during sampling [2, 5, 6, 13–17], in Rwanda the TB prevalence rate was as per expectation lower than estimated previously by WHO. This lower TB burden will be the basis for all future TB control activities.

Males were more likely to have TB than females, something also observed in other countries [2, 13, 15–17]. The survey indicated TB in males to be 5 times that of in females while in the routine surveillance data this is only twice as high, suggesting males are still being underdiagnosed in Rwanda. Previous literature from the country suggested that the observed difference in routine data was due to the lower sensitivity of TB screening and diagnostic tools in females compared to males [18]. The current survey data indicate that this observed difference between males and females is true and not a limitation of the TB detection system to diagnose TB in females. The difference may then be attributed to either intrinsic (biological difference) or extrinsic factors. Estrogens may have an immune enhancing effect, while progesterone and androgens may have an immune suppressive effect [19]. Risk factors such as repetitive TB infections, malnutrition, HIV infection, smoking, harmful alcohol use, indoor air pollution, that are more prevalent among males could also underlie the observed difference. In fact, this survey revealed that males are three times more likely to smoke than females and reported drinking alcohol twice as often compared to females [20]. In Rwanda, HIV infection and low body mass index seem not to be an explanation as they are more prevalent in females than males [21].

Older people were clearly more affected by TB than younger, with a gradually increasing TB burden with increasing age. This relation has also been observed in national TB prevalence surveys in other countries [2, 5, 13, 15]. This points to an ageing TB epidemic in Rwanda, suggesting new transmissions are declining and cases occurring from past rather than recent TB infections [22].

Chest x-ray screening was more predictive for TB diagnosis than symptoms screening based on cough of any duration. This was also observed in other surveys [15, 22]. At the time of survey, symptom screening in the routine program was based on cough of two weeks or more, while chest x-ray was not used as routine screening tool.

The survey resulted in a much lower number of cases detected than anticipated. An important question is whether TB cases have been missed during the conduct of the survey. Reviewing this, there are an indication that only a few cases could have been missed during field operations for which during analysis adjustment was largely done through missing value imputation. First, participation rate was very high in Rwanda, much higher than in most other countries who recently completed TB prevalence surveys [2, 5, 6, 13–17]. Second very few people were not administered both screening tools, only 7 missed the symptoms screen and 68 (of which 10 had cough) did not undergo CXR screening. Third, quality indicators for chest x-ray indicated results are reliable as only 0.2% of all CXR images reread were identified as abnormal suggestive TB at central level while scored in the field as normal. Culture recovery was good in the Rwanda survey at 88% suggesting culture was performed well which was also supported by EQA reports.

At the time of survey design and implementation in Rwanda, the guidance was to perform sputum smear and culture as per the WHO guideline [10]. Subsequent surveys like for example the TB prevalence survey in Zambia added molecular testing by the Xpert MTB/RIF assay to confirm MTB positivity for all smear positives cases, to test samples of cases with culture indeterminate and to test samples of cases with CXR suggestive of TB but without bacteriological indication of MTB in smear or culture [17]. In the Zambia survey, 17 additional cases (6% of all survey cases) were detected based on Xpert MTB/RIF assay [17]. Extrapolating this to the Rwanda results, would yield two additional cases (6% of 40 survey cases) that potentially have been missed by not performing Xpert MTB/RIF. Therefore, the Rwanda survey final result might have not been substantially different if the Xpert MTB/RIF assay has been performed.

As limitation, this survey excluded displaced people living in camps, military barracks, prisoners and boarding schools as guided by the handbook of TB prevalence survey and currently the effect of these groups is unknown. In addition, children were not included due to the difficult diagnostic of pulmonary TB diagnostic among children.

In conclusion, the first direct measure of Rwanda’s TB prevalence was lower than that estimated by WHO. A low burden makes it more challenging to detect and treat the remaining cases. Rwanda has to maintain routine detection strategies for the general population while at the same time new and more active case detection strategies need to be implemented targeting high risk populations. Based on the survey findings, Rwanda adopted more sensitive screening approaches (any cough and x-ray screening), and more sensitive diagnostic approaches (GeneXpert as initial test), for identified key groups of the population at higher risk of developing TB disease like those 55+ and contact of TB index cases [23].

## Supporting information

S1 Data (DOCX)
