# Supplementary material for: Prevalence of tuberculosis in Rwanda: Results of the first nationwide survey in 2012 yielded important lessons for TB control
Source: PLoS One. 2020 Apr 23;15(4):e0231372. doi: 10.1371/journal.pone.0231372 (PMC7179849; doi:10.1371/journal.pone.0231372)
Supplement: S1 Data — (DOCX) [file pone.0231372.s001.docx]

Form n^o^: 03

| **Cluster N^o^** | **House Hold N^o^** | **Participant N^o^** |
| --- | --- | --- |
|  |  |  |

# Screening questionnaire (annex 5)

Names: _____________________________________ Age ______ Sex: M □ F □

Village :_____________________

**DATE OF INTERVIEW: ___ / ___ / _____** Code of interviewer: __________________

**Je voudrais vous poser quelques questions dans votre santé.**

***Nashakaga kubabaza ibibazo bimwe na bimwe ku buzima bwanyu***

1. Êtes-vous sous traitement anti TB actuellement? **Oui Non Inconnu**

***Mwaba muri ku miti ivura igituntu***

1. Avez- vous été sous traitement anti TB avant? **Oui Non Inconnu**

***Mwaba mwarigeze kujya ku miti ivura igituntu***

1. Avez-vous de la toux ? ***si non passer a la question 7* Oui Non Inconnu**

***Mwaba mufite inkorora***

1. Votre toux dure depuis 2 semaines ou plus? **Oui Non Inconnu**

***Inkorora yanyu yaba imaze ibyumweru 2 cg birenga***

1. Votre toux est-elle productive d’un crachat? **Oui Non Inconnu**

***Inkorora yanyu yaba itanga igikororwa***

***si non passer a la question 7***

1. Votre crachat est-il strié de sang/hemoptoique?(***si non passer a la question 7***) **Oui Non Inconnu**

***Igikororwa cyanyu cyaba kiba kirimo amaraso***

1. Avez-vous de la fièvre ? **Oui Non Inconnu**

***Mugira umuriro***

1. Avez-vous constaté une perte pondérale inattendue **Oui Non Inconnu**

durant ces 4 dernières semaines ou plus?

***Mwaba mwaratakaje cyane ibiro muri ibi byumweru 4 bishize***

1. Avez-vous constaté des sueurs nocturnes Abondante ? **Oui Non Inconnu**

***Mwaba mututubikana cyane mu ijoro***

**SCRNG RESULT**

| Si la réponse a la question **3. est oui, et/ou si la réponse est oui a au moins deux des 7, 8 ou 9,** alors le sujet est un suspect de TB potentiel. |
| --- |
